# Assessing burden, risk factors, and perceived impact of uterine fibroids on women’s lives in rural Haiti: implications for advancing a health equity agenda, a mixed methods study

**DOI:** 10.1186/s12939-020-01327-9

**Published:** 2021-01-01

**Authors:** Christophe Millien, Anatole Manzi, Arlene M. Katz, Hannah Gilbert, Mary C. Smith Fawzi, Paul E. Farmer, Joia Mukherjee

**Affiliations:** 1 Partners In Health, Boston, MA USA; 2 Zanmi Lasante, Mirebalais University Hospital (MUH), Route Chatulee, Mirebalais, HT5211 Haïti; 3 Department of Global Health and Social Medicine, Harvard Medical School, Boston, MA USA; 4 Division of Global Health Equity, Brigham and Women’s Hospital, Boston, MA USA

**Keywords:** Uterine fibroids, Risk factors, Women, Poverty, Haiti

## Abstract

**Background:**

Uterine fibroids, the most common cause of gynecologic surgery, have a reported cumulative incidence of 59% among Black women in the U.S. Uterine fibroids negatively impact the quality of women’s lives. No study has been found in the literature about fibroids in Haiti. We conducted a mixed methods study to assess the burden and risk factors of uterine fibroids, as well as their effects on women’s quality of life.

**Methods:**

A convergent mixed methods study was conducted between October 1, 2019 and January 31, 2020 at MUH’s (Mirebalais University Hospital) OB-GYN outpatient department. Quantitatively, in a cross-sectional study 211 women completed consecutively a structured questionnaire. In-depth interviews with 17 women with fibroids and 7 family members were implemented for the qualitative component. Descriptive statistics were calculated for clinical and social demographic variables. Logistic regression was performed to examine associations between fibroids and related risk factors. An inductive thematic process was used to analyze the qualitative data. A joint display technique was used to integrate the results.

**Results:**

Of 193 women analyzed 116 had fibroids (60.1%). The mean age was 41.3. Anemia was the most frequent complication— 61 (52.6%). Compared to women without uterine fibroids, factors associated with uterine fibroids included income decline (AOR = 4.7, 95% CI: 2.1–10.9, p = < 0.001), excessive expenses for transport (AOR = 4.4, 95% CI: 1.6–12.4, *p* = 0.005), and family history with uterine fibroids (AOR = 4.6, 95% CI: 1.6–13.6, p = 0.005). In contrast, higher level of education and micro polycystic ovarian syndrome were associated with lower prevalence (AOR = 0.3, 95% CI: 0.1–0.9, *p* = 0.021) and (AOR = 0.2, 95% CI: 0.1–0.97, *p* = 0.044), respectively. The qualitative findings delineate how contextual factors such as health system failures, long wait times, gender inequality and poverty negatively affect the quality of women’s lives. The poverty cycle of uterine fibroids emerged.

**Conclusions:**

A vicious cycle of poverty negatively impacts access to care for uterine fibroids in Haiti. Health insurance, social support, and income generating activities may be keys to promote social justice through access to adequate care for women with uterine fibroids in Haiti.

## Background

Uterine fibroids are benign tumors of the smooth muscle of the uterus and their burden across populations varies widely – with rates ranging from 4.5 to 68.6% [1]. Baird & all’s study indicated that cumulative incidence of premenopausal women with uterine fibroids by ultrasound screening was 59% among Black women compared to 43% among white women in the U.S [2]. The same study reported rates of uterine fibroids as high as greater than 80 and 70%, among Black and white women, respectively, by the age of fifty [2]. In Haiti as it is the case in other populations the rates of uterine fibroids have not been documented.

Studies have identified a variety of risk factors for fibroids including age, race, family history, and comorbid conditions such as diabetes and hypertension [1, 3]. Clinical manifestations of fibroids can range from asymptomatic presentations to more severe conditions such as bleeding, dysmenorrhea, and fertility problems [1, 4–6]. Fibroids – when left untreated – are associated with a number of debilitating complications such as acute or chronic pelvic pain, anemia, pyomyoma, hydronephrosis, and premature delivery as well as fetal intra-uterine growth retardation [7–11].

Fibroids are the most common benign tumors of the uterus and the main indication of surgery in gynecology [12]. Without treatment this condition leads to significant chronic morbidity - and even mortality. In communities that lack sufficient access to medical care, uterine fibroids have been shown to deeply affect a woman’s quality of life [13].

There are several methods of treatment currently available for uterine fibroids – including radiological, medical, and surgical interventions [12, 14]. High-resource countries have achieved remarkable improvements in the treatment of uterine fibroids, including the emergence of laparotomy, laparoscopy and robotic surgery [12, 14, 15]. These treatment options available to women in these settings have helped many women conserve their fertility. In contrast, access to modern fibroid surgical care is largely out of reach for most women in low and middle income settings because the medical equipment, infrastructure and specialized personnel required for such interventions are not widely available [16–19].

In Haiti, a low-income country, there are only seven beds per 10,000 people compared to the Dominican Republic its neighboring country with 16 beds per 10,000 people according to the WHO website consulted June 22, 2020. The availability of specialized care is even more dire, as Haiti only has 5.9 surgical specialists including obstetricians, surgeons, and anesthetists per 100,000 people [20], in marked contrast to the 20–40 anesthetists, and obstetrics-gynecology (OB-GYN) specialists per 100,000 people recommended by the Lancet Commission for global surgery [18]. In addition, the lack of equipped operating rooms, medicine, and other human resources hamper access to care for uterine fibroids. Further, the majority of Haitians live in rural areas and have limited access to basic health care and even less access to surgical care [21].

To assess the current epidemiology and effects of uterine fibroids on the quality of women’s lives in Haiti, we conducted a convergent mixed methods study – the first of its kind. We implemented a cross sectional study to examine the prevalence, complications, and risk factors associated with uterine fibroids. We also conducted a qualitative study to explore patients’ illness experience and care seeking trajectories, to understand the impact of uterine fibroids, and identify the structural barriers that shape access to fibroid care.

## Methods

### Study setting

Mirebalais University Hospital (MUH) is a three-hundred bed facility located in the Central Plateau built and supported by Partners In Health in collaboration with the government of Haiti. One hundred of these beds are in the OB-GYN Department. MUH covers two catchment areas including Mirebalais, Savanette, Saut d’Eau with 187,077 people and North, Northeast, Artibonite, part of the west department which serve more than 3.1 million people [22]. While MUH has only 6 operating rooms, over 6000 surgeries are performed every year. Approximately 250,000 patients visit MUH every year. Of all surgical interventions, 40% are for OB-GYN conditions while 12% are related to GYN conditions identified during outpatient visits.. The OB-GYN service includes a triage area, labor and delivery, pre- and post-partum wards, and one gynecology ward. The hospital offers a large range of women’s health services including prenatal care, family planning, ultrasound, gynecological care, complete emergency obstetrical care, cancer screening, and gynecological surgery (including uterine fibroids).

Uterine fibroids represent the most common illness among women presenting at the MUH’s outpatient department (OPD) at the OB-GYN ward.

### Study design

This study used a convergent mixed methods design in which qualitative data were collected to provide elaboration, clarification, and explanation for the quantitative results and vice versa [23]. The quantitative cross-sectional study included all women 20 years of age or older who were seeking gynecological care at MUH’s outpatient gynecology ward from October 1, 2019 to January 31, 2020. Semi-structured interviews included 17 women with uterine fibroids and 7 of their family members. Purposeful sampling [24] was used to identify both sets of participants and to maximize variation. Participants were selected in relatively equal numbers from within and outside of the Central Department.

Women having current gynecologic ultrasound results documented in their medical records during the study period were invited to participate in the study. Family members who knew and supported the participants over time with the disease were selected based on their consent and the participant’s reference to the research team.

Excluded from the study were women who: were less than 20 years old, presenting for prenatal care at MUH, and those who refused to consent. Further, women who had a medical emergency or who otherwise were not able to participate due to medical considerations were not included in the study.

### Measures

#### Quantitative data collection

**Fig. 1:**
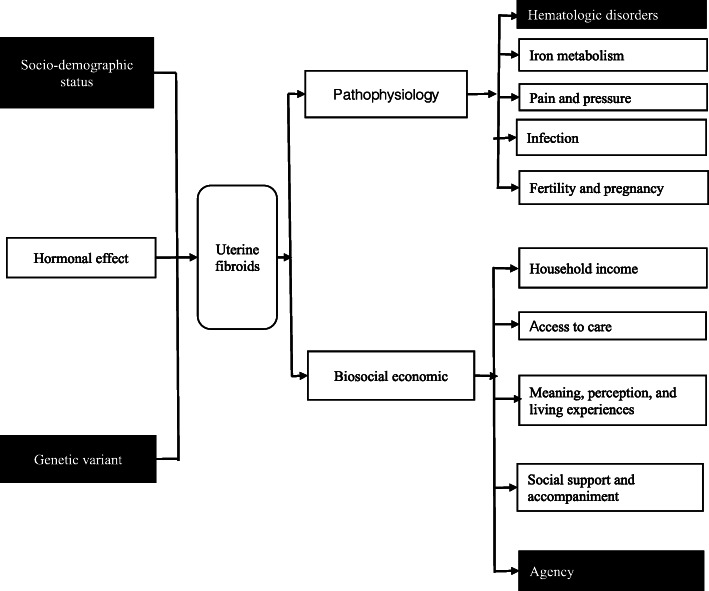
Conceptual framework

The study instruments were validated after being tested during the first week on patients at the OPD of the OBGYN department to observe potential misunderstanding among participants. A conceptual framework (Fig. 1) was used to inform the survey questionnaire which included important covariates including age, parity (number of births), menopause, patient status (initial or follow-up visit), confirmed diagnosis of uterine fibroids, employment, monthly income was assessed by asking the participants to estimate their monthly income, income decline was assessed by asking the participants if they observed any decline in their monthly income after having the diagnosis of uterine fibroids, excessive expenses were assessed by capturing any out pocket expenses that limit their ability to meet the basic needs such as tuition fees for their children, groceries, transport, etc. while seeking care for uterine fibroids, household expenses (such as education, food, self-care, and medical care, etc.), zone of residence, average time to get to the hospital, primary means of transport, main profession, health insurance status, education level, and family conflicts. Family conflict was assessed by reported problems of communication and disagreements between the women and their partners after the diagnosis of uterine fibroids [25]. The most frequent clinical signs and symptoms associated with uterine fibroids were ascertained including constipation, dysmenorrhea (pain during menstruation), acute pelvic pain (less than 3 months), chronic pelvic pain (more than 3 months), menorrhagia (abundant bleeding during menstruation), metromenorrhagia (bleeding between periods), mictional difficulty (pain with urination), pelvic pressure, pelvic infection, infertility (defined as > 1 year without conceiving a child while being sexually active and not receiving family planning); related conditions included deep vein thrombosis, urinary stones, hydronephrosis, and anemia (Hb < 12 mg/dl) [6, 9, 11, 15, 26]. We also measured self-reported stress and depression using a locally validated depression screening tool, the Zanmi Lasante-Depression Symptom Inventory (ZL-DSI) (no depression < 13, mild depression 13–17, moderate depression 18 to 27, and severe depression 28–39) [27]. Demographics and clinical symptoms data were obtained using a structured questionnaire. We performed chart reviews to extract data on clinical diagnosis of uterine fibroids and complications of uterine fibroids.

#### Qualitative data collection

Participants in the qualitative study took part in a single, in-depth semi-structured interview which lasted 60–90 min. Interviews took place in a confidential and quiet room prepared by the study team. All interviews were conducted in Haitian Creole. An experienced researcher was trained to serve as a data collector a long with the principal investigator. Semi-structured interview guides were used to capture data on the following topics: [a] experiences of living with fibroids; (b) their care-seeking trajectory; (c) the meaning attributed to fibroids; and (d) the effect of fibroids on the family’s social and economic life.

### Data analysis

Descriptive statistics were used to report the sociodemographic and clinical characteristics of uterine fibroids. Chi-square and Fisher’s exact tests were performed to generate *p*-values for comparing the sociodemographic and clinical characteristics for both groups. Fisher’s exact test was used when cell counts were less than 5. Bivariate and multivariate logistic regression were used to identify the risk factors associated with uterine fibroids. We used stepwise regression to build our model. We retained variables that were differentially distributed among cases with uterine fibroids (*p* < 0.2 in the design-adjusted Chi-squared). Collinearity was assessed, and for covariates that were identified to be strongly collinear (r > =0.8, using Pearson’s correlation test), the variable more strongly correlated with uterine fibroids or those known as important were retained for model building [28]. All quantitative analyses were performed using Stata, version 16.2.

**Fig. 2:**
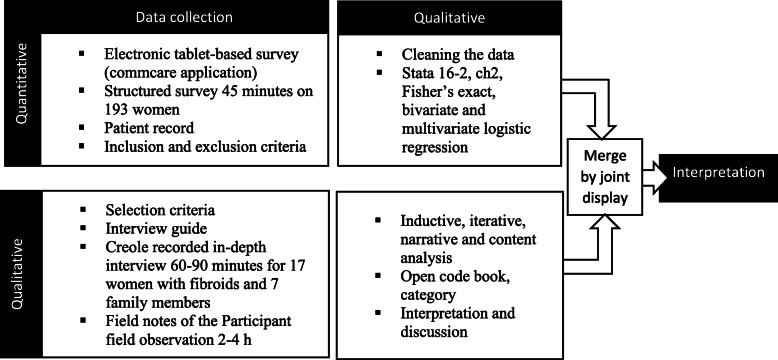
Summary of the convergent mixed methods design

For the qualitative analysis, a narrative analysis of all transcripts was performed to identify key concepts or theories related to the research question. Then, transcripts were analyzed in depth using an inductive, narrative and thematic content analysis approach [29, 30]. Transcripts were reviewed in full, and a subset of transcripts were open-coded by the first author to identify a set of emerging concepts that explained the perceived impacts of uterine fibroids. These initial concepts were reviewed by the third and the fourth authors, and subsequently revised to develop a draft codebook. The codebook was piloted and revised into a final codebook which was in turn used to code the entire dataset. The coded data was analyzed using an inductive process that sought to identify a set of key descriptive concepts. These initial concepts were developed by the first author and revised by the third and fourth authors through an iterative approach to the data, resulting in a final set of descriptive categories (Fig. 2).

Finally, quantitative and qualitative results were integrated by using a joint display technique [31] – the ‘Poverty cycle’ - which highlights the interconnected phenomena emerging from the qualitative and quantitative results (Fig. 4). This figure identifies points of convergence across the qualitative and quantitative datasets while demonstrating the cyclical processes that marked the experience of women living with fibroids in rural Haiti.

## Results

### Quantitative findings

**Fig. 3:**
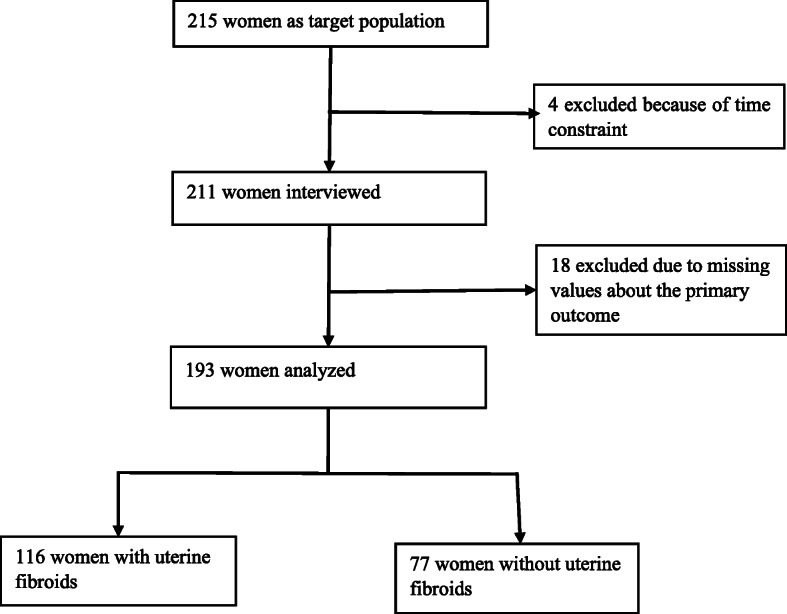
Data flow

The data flow of all participants in the study is described in the Fig. 3.

Among the populations studied, 70 (60.3%) were between 35 to 49 years old. Sixty-one (52.6%) were from the Central plateau Department (primary catchment area), whereas 55 (47.4%) were from other areas of the country. Over one hundred (87.1%) did not have health insurance. Sixty-seven (57.8%) reported that they experienced a decline in household income. Table 1 describes demographic characteristics and complications of uterine fibroids of study participants.

**Table 1:** Sociodemographics, economic characteristics, and clinical history of women with and without uterine fibroids looking for care at Mirebalais University Hospital’s outpatient department between October first, 2019 to January 31, 2020

	Fibroids (Yes)(n = 116)	Fibroids (No) (***n*** = 77)	***P*** value
characteristics	n (%) or mean	n (%) or mean	
Age (mean)	41.3 (20–77)	39.4 (22–75)	0.490
Age category			0.013
20–34	28 (24.1)	31 (40.3)	
35–49	70 (60.3)	30 (38.9)	
50+	18 (15.5)	16 (20.8)	
Number of pregnancies			0.387
No pregnancy	28 (24.1)	14 (18.2)	
1–3 pregnancies	52 (44.8)	42 (54.5)	
> 3 pregnancies	20 (17.2)	11 (14.3)	
Number of births			0.210
No childbirth	43 (37.1)	22 (28.6)	
1–3 childbirths	47 (40.5)	41 (53.2)	
> 3 childbirths	11 (9.5)	5 (6.5)	
Menopause			0.550
No	81 (69.8)	57 (74.0)	
Yes	20 (17.2)	11 (14.3)	
Family history of fibroids			< 0.001
No	25 (21.6)	22 (28.6)	
Yes	58 (50.0)	10 (12.9)	
Patient status			0.021
Follow-up visit	95 (81.9)	72 (93.5)	
Initial visit	21 (18.1)	5 (6.5)	
If follow-up patient			0.051
< 6 months	8 (5.7)	3 (3.9)	
6–12 months	15 (9.8)	4 (5.2)	
> 12 months	72 (70.9)	65 (84.4)	
Zone of Residence			0.009
Centre	61 (52.6)	55 (71.4)	
Out of central plateau	55 (47.41)	22 (28.6)	
Type of residence			0.266
Urban	91 (78.5)	55 (71.4)	
Rural	25 (21.6)	22 (28.6)	
Average time per medical appointment/visit (Time to go to the hospital)			0.285
≤ 2 h	92 (79.3)	65 (84.4)	
> 2 h	22 (18.9)	10 (12.9)	
Means of transport			0.016
Public transport	100 (87.1)	55 (71.4)	
Walk	10 (8.6)	17 (22.1)	
Private transport	4 (3.5)	5 (6.5)	
Main profession			0.137*
Small business /market	48 (41.4)	30 (38.9)	
Educator	15 (12.9)	7 (9.1)	
Nurse	13 (11.2)	14 (18.2)	
Farmer	12 (10.3)	2 (2.6)	
Other	28 (24.1)	24 (31.2)	
Health insurance			0.094
No	101 (87.1)	60 (77.9)	
Yes	5 (12.9)	17 (22.1)	
Education			0.036
Primary	27 (23.3)	9 (11.7)	
Secondary or higher	74 (63.8)	59 (76.6)	
Family conflicts			0.023
No	86 (74.1)	67 (87.0)	
Yes	27 (23.3)	8 (10.4)	
Employment status			0.744
Not employed	69 (59.5)	47 (61.0)	
Employed	47 (40.5)	29 (37.7)	
Monthly income			0.255
15.4 USD - ≤206 USD (≤10 USD/day)	102 (87.9)	73 (94.8)	
> 206USD (> 10 USD/day)	11 (9.5)	4 (5.2)	
Income decline (decline observed in the income after the diagnosis of uterine fibroids)			< 0.001
No	49 (42.2)	53 (68.8)	
yes	67 (57.8)	24 (31.2)	
Degree of income decline if income decline observed			0.560*
High	49 (61.5)	1 (1.3)	
Moderate	21 (32.3)	5 (6.5)	
Low	4 (6.2)	18 (23.4)	
Major expense categories			0.529
Medical care	23 (19.8)	12 (15.6)	
Non-medical care	93 (80.2)	65 (84.4)	
Excessive transport expenses			0.001
No	10 (8.6)	20 (25.9)	
Yes	91 (78.4)	48 (62.3)	
**Clinical diagnostics and complications**			
Diagnostic of uterine fibroids			
No	0 (0.0)	77 (39.9)	
Yes	116 (60.1)	0 (0.0)	
Anemia			0.789
No	55 (47.4)	35 (45.4)	
Yes	61 (52.6)	42 (54.6)	
Infertility			0.197
No	73 (62.9)	57 (74.0)	
Yes	39 (33.6)	20 (25.9)	
Deep vein thrombosis			0.001
No	97 (83.6)	76 (98.7)	
Yes	18 (15.5)	1 (1.3)	
Urinary stone			0.972
No	99 (85.3)	65 (84.4)	
Yes	17 (14.7)	11 (14.3)	
Hydronephrosis			0.246*
No	110 (94.8)	76 (98.7)	
Yes	6 (5.2)	1 (1.3)	
Pelvic infection			0.168
No	31 (26.7)	14 (18.2)	
Yes	82 (70.7)	61 (79.2)	

*p-values generated from Fisher’s exact test for cell counts less than 5

Of 193 participants included in the analysis, 116 (60.1%) had uterine fibroids (Table 1). The two most frequently observed clinical symptoms included stress 92 (79.3%) and dysmenorrhea 73 (62.9%) (Table 2). In addition, documented anemia 61 (52.6%), and infertility 39 (33.6%) were the two most prevalent major complications (Table 1).

**Table 2:** clinical Signs and symptoms of women with uterine fibroids in Haiti accessing care at the outpatient department at Mirebalais University Hospital (*n* = 116)

Characteristics	n (%) or mean
Stress (*n* = 114)	92 (79.3)
Dysmenorrhea (115)	73 (62.9)
Acute pelvic pain (112)	72 (62.1)
Pelvic mass (*n* = 113)	56 (48.3)
Polymenorrhagia	50 (43.1)
Metrorrhagia (115)	49 (42.2)
Menorrhagia (n = 114)	49 (40.5)
Mictional difficulty	49 (40.5)
Chronic pelvic pain (*n* = 115)	33 (28.5)
Pelvic pressure (n = 114)	33 (28.5)
ZLDSI score (mean)	7.65 (0–24)
Low depression (ZL score 13–17 and ≥ 18 - ≤ 27)	22 (11.4)
Abdominal pelvic mass (n = 113)	9 (7.8)
Moderate depression (≥18 - ≤ 27)	8 (4.2)

In the bivariate analysis (Table 3, model 1.), the odds of having uterine fibroids were 3.8 times greater among those who experienced excessive expense for transport compared to those who did not have this expense (95% CI: 1.65–8.74, *p* = 0.002). In addition, those who experienced income decline had a 3-fold greater odds of having uterine fibroids compared to those who did not (95% CI: 1.64–5.54, *p* < 0.001), and those who were farmers had a 5.1-fold greater risk of having uterine fibroids compared to those without a specific profession (95% CI: 1.04–25.29, *p* = 0.044). Family history with fibroids also demonstrated a strong association with uterine fibroids in this population (OR = 5.1, 95% CI: 2.11–12.33, *p* < 0.001).

**Table 3:** Bivariate (model 1.) and multivariate (model 2.) analysis examining risk factors associated with uterine fibroids among sociodemographic and clinical variables at MUH age greater or equal to 20 years old (*n* = 193)

Model 1. Bivariate analysis			
**Sociodemographic characteristics**	**Odd ratio**	**95% CI**	**P-value**
Age category			
20–34	–		
> 35–49	2.6	(1.33–5.03)	0.005
50+	1.2	(0.53–2.90)	0.611
Zone of residence			
Central plateau	–		
Out of central plateau	2.3	(1.22–4.17)	0.01
Number of pregnancies			
No pregnancy	–		
1–3 pregnancies	0.6	(0.29–1.32)	0.216
> 3 pregnancies	0.9	(0.34–2.41)	0.848
Number of births (101)			
No childbirth	–		
1–3 childbirths	0.6	(0.30–1.14)	0.115
> 3 childbirths	1.1	(0.35–3.64)	0.844
Menopause			
No	–		
Yes	1.3	(0.57–2.88)	0.551
Patient status			
New patient	–		
Follow up	3.2	(1.14–8.84)	0.026
If follow-up patient			
< 6 months	–		
6–12 moths	1.4	(0.25–7.89)	0.699
≥ 12 months	0.4	(0.11–1.63)	0.208
Type of residence			
Rural	–		
Urban	1.5	(0.75–2.83)	0.267
Average time per medical appointment			
≤ 2 h	–		
> 2 h	1.6	(0.69–3.50)	0.287
Excessive expense for transport			
No	–		
Yes	3.8	(1.65–8.74)	0.002
Means of transport			
Walk	–		
Public transport	3.1	(1.34–7.28)	0.008
Private transport	1.4	(0.29–6.27)	0.694
Health insurance			
No	–		
Yes	0.5	(0.24–1.12)	0.098
Education level			
Primary	–		
Higher education	0.4	(0.18–0.957)	0.039
Main profession			
Other (no specific profession)	–		–
Small business	1.4	(0.67–2.79)	0.384
Educator	1.8	(0.64–5.24)	0.256
Farmer	5.1	(1.04–25.29)	0.044
Nurse	0.8	(0.31–2.02)	0.631
Employment			
No	–		
Yes	1.1	(0.61–1.99)	0.744
Monthly Income			
15 USD- ≤206USD (≤10 USD/day)	–		
> 206USD (> 10 USD/day)	1.9	(0.60–6.42)	0.262
Income decline (decline observed in the income after the diagnosis of uterine fibroids)			
No	–		
Yes	3.0	(1.64–5.54)	< 0.001
Degree of income decline			
Low	–		
Moderate	1.1	(0.09–11.56)	0.968
High	0.6	(0.06–5.33)	0.610
Expenses category			
Medical	–		
Non-medical	0.7	(0.35–1.60)	0.455
**Clinical and psychosocial characteristics**			
Family conflicts			
No	–		
Yes	2.6	(1.12–6.16)	0.026
Family history with fibroids			
No	–		
Yes	5.1	(2.11–12.33)	< 0.001
Pelvic infection			
No	–		
Yes	0.6	(0.30–1.24)	0.170
Ovarian micro polycystic			
No	–		
Yes	0.2	(0.12–0.41)	< 0.001
Constipation			
No	–		
Yes	1.6	(0.80–3.05)	0.191
Pelvic mass			
No	–		
Yes	2.0	(1.10–3.67)	0.024
Abdominal-pelvic mass			
No	–		
Yes	1.6	(0.46–5.25)	0.475
Mictional difficulty			
No	–		
Yes	1.7	(0.92–3.16)	0.091
Pelvic pressure			
No	–		
Yes	1.5	(0.77–3.02)	0.225
Acute pelvic			
No	–		
Yes	1.75	(0.97–3.17)	0.062
Chronic pelvic pain			
No	–		
Yes	1.3	(0.65–2.48)	0.476
Polymenorrhagia			
No	–		
Yes	1.6	(0.89–3.04)	0.111
Menorrhagia			
No	–		
Yes	1.6	(0.89–3.04)	0.111
Metrorrhagia			
No	–		
Yes	1.6	(0.89–3.01)	0.111
Dysmenorrhea			
No	–		
Yes	1.6	(0.89–2.89)	0.113
Infertility			
No	–		
Yes	1.5	(0.80–2.89)	0.198
Stress			
No	–		
Yes	1.4	(0.68–2.75)	0.376
Anemia			
No	–		
Yes	0.9	(0.52–1.64)	0.789
Deep vein thrombosis			
No	–		
Yes	14.1	(1.84–108.02)	0.011
Urinary stone			
No	–		
Yes	1.0	(0.45–2.30)	0.972
Hydronephrosis			
No	–		
Yes	4.1	(0.49–35.13)	0.192
Depression			
No	–		
low (score 13–17)	1.6	(0.60–4.06)	0.350
Moderate (score 18–24)		(0.62–42.73)	0.130
**Model 2. Multivariate analysis**			
**Characteristics**	**Adjusted OR**	**95% CI**	**P-value**
Age category			
< 35	–		
35–49	1.7	(0.76–4.02)	0.189
50–77	0.4	(0.11–1.50)	0.174
Patient status			
Initial Visit	–		
Follow -up visit	2.8	(0.79–9.95)	0.110
Excessive expense for transport			
No	–		
Yes	4.4	(1.55–12.38)	0.005
Heath Insurance			
No	–		
Yes	1.2	(0.44–3.30)	0.709
Education level			
Primary	–		
Higher education	0.3	(0.09–0.87)	0.021
Income decline(decline observed in the income after the diagnosis of uterine fibroids)			
No	–		
Yes	4.7	(2.05–10.93)	< 0.001
Number of births			
No childbirth	–		
1–3 childbirths	0.6	(0.26–1.30)	0.188
4+ childbirths	0.9	(0.19–3.99)	0.866
Family conflicts			
No	–		
Yes	3.1	(0.54–17.9)	0.206
Family history of fibroids			
No	–		
Yes	4.6	(1.58–13.56)	0.005
Pelvic infection			
No	–		
Yes	0.4	(0.09–1.80)	0.233
Micro polycystic ovary			
No	–		
Yes	0.3	(0.10–0.97)	0.044
Pelvic Mass			
No	–		
Yes	2.2	(0.72–6.85)	0.164
Frequent menstruation			
No	–		
Yes	0.4	(0.14–1.16)	0.094
Deep Vein thrombosis			
No	–		
Yes	3.0	(0.29–31.89)	0.352
Depression			
No	–		
Low	5.7	(0.56–59.18)	0.142
Moderate	2.4	(0.17–36.03)	0.513

In the multivariate analysis women who experienced excessive expenses for transport had a 4.4 times greater adjusted odds of having uterine fibroids compared to those who did not experience excessive expense for transport (95% CI: 1.55–12.38, *p* = 0.005). In contrast, women with higher education were less likely to be diagnosed with uterine fibroids compared to those with lower education (AOR = 0.3, 95% CI: 0.09–0.87, *p* = 0.021). Women with income decline had a 4.7 times greater adjusted odds of uterine fibroids compared to those who did not experience an income decline (95% CI: 2.05–10.93, p < 0.001). In terms of clinical factors, women with family history of uterine fibroids had a 4.6 times greater adjusted odds of having uterine fibroids compared to those with no family history of uterine fibroids (95% CI: 1.58–13.56, p = 0.005). The condition of micro polycystic ovary is less likely to be observed among women with uterine fibroids compared to those without uterine fibroids (AOR = 0.3, 95% CI: 0.10–0.97, *p* = 0.044). Associations were not observed between age, patient status, health insurance, number of births, family conflict, polymenorrhagia, deep vein thrombosis, or depression categories with uterine fibroids in the multivariate model.

### Qualitative findings

Qualitative data for seventeen women with uterine fibroids and seven family members of these women were analyzed. Four key themes (A-D, below) describing women’s experiences living with fibroids were inductively identified. They describe the complications and consequences of women’s care-seeking trajectories and highlight the structural and contextual factors that shape them.

#### A. Health system failure

Many women reported in their interviews that the Haitian health system did not deliver adequate care for their fibroids. While some participants indicated that they began their care-seeking by consulting with a traditional healer close to their home, most eventually sought care for their symptoms within the public or private health system. Women explained that before arriving at MUH, they undertook an extensive number of visits to different care providers – “roaming” from one hospital or clinic to the next in an attempt to seek care for their condition. They recounted that at each stage they were unable to obtain effective treatment. For many, the protracted search for care led to significant delays, increasing pain, and mounting health care costs.

“I came to know I had fibroids at General Hospital in Port-Au-Prince. [Before that] the « Medsen Fey » [herbalist and shaman] told me I was pregnant. Well, there was a « Medsen Fey » who told me I was going to have twins. I said, ‘God knows everything. I know nothing.’ I spent money over, over, and over again [on treatments] and nothing worked out. I got really sick on July 26, 2019. They rushed me to the emergency room of the General Hospital. I came here [to MUH] after I left the emergency room of General Hospital. They asked me to bring the sonography result back, but I could not find a doctor. I finally found one at 9: 00 a.m. I was sent to *Rue Monseigneur Guilloux* [private clinic]. And … it was 10: 00 a.m. When I got to the place I was sent, the receptionist told me, ‘No, there is no doctor here for this disease’ and said I have to come back the following day at 6: 00 a.m. My cousin could not come with me because she had to drop her children off to school. My other cousin told me she would come to take me and bring me here. They came here with me. General Hospital did not transfer me to MUH. My cousins and I came here ourselves. I consulted several times”.

-Unemployed woman with uterine fibroids from Port-Au-Prince.

#### B. Long wait time for incomplete services

Traveling to MUH Mirebalais from the Central Plateau via public transport often entailed a long wait time for getting access to care for their fibroids. Women explained that they often had to leave their homes a full day before their medical appointment. They noted that seeking care for their fibroids at MUH required reporting to different services within the hospital, and each service had a long queue. In some cases, participants reported spending an entire day waiting for their medical consultation, only to be told to leave the hospital and return on the next day. For these women, the wait for a single consultation resulted in a long time commitment with significant social and economic implications.

“I usually leave home at 6:00 am… I take the bus in Delmas 33 to go to Croix-Des-Bouquets. I take another bus there so that I get to the hospital by 8:00 a.m. Well…when I get there, I have to get my records released, I have to get my vital signs checked and, they transfer my records to the doctor. I wait for the doctor if he has not arrived yet… There are a lot of people. There are a lot of people [waiting] for gynecology. This process is very long … I do not like to sleep at the hospital. There is nowhere to sleep there. If I do a test today at the hospital, I must come take the results tomorrow. So, I go [home] to Port-Au-Prince and I return back to Mirebalais. So, that costs me money.”

-Teacher with uterine fibroids from Port-au-Prince.

#### C. Gender inequality

Women with uterine fibroids explained that they often felt pressured to perform all housework including cooking and caring for children (if applicable) with little to no support or minimal support from their spouses or partners. Family members shared this same perception. The effects of uterine fibroids made it exceedingly difficult for women to carry out the physical labor expected of them, but they nonetheless attempted to keep up with the duties they were expected to fulfill.

“Usually, I do everything, I wash clothes, I make sure that my husband’s clothes are ready, I make sure that my child’s clothes are ready, I make sure they have food and I give the maid instructions while I am not at home.”

-Nurse with uterine fibroids from Delmas.

“If she [my wife] was in good health, she would do all the housework. But she cannot. She cannot take care of the household. Sometimes she cooks, but sometimes she does not. She cannot sit and cook when she suffers from the complications of the disease.”

-Family member, husband from Mariani.

Women reported that the effects of the gendered expectations related to fertility were particularly painful for them. Fertility challenges caused women personal sadness, but infertility also led to social exclusion. Women explained that that they were blamed for the fertility challenges that they experienced because of untreated fibroids:

“I am unhappy and so is my husband because of the fibroid, because most Haitian men would like to have children. As soon as you cannot, their family members start naming you: “*Manman Milet*” (a sterile woman).”

-Teacher with uterine Fibroids from Mirebalais.

#### D. Poverty

Women indicated in their interviews that the physical effects of fibroids imposed a number of social and economic consequences on them. Notably, women with fibroids were largely excluded from social and economic opportunities. Participants recounted how the effects of their fibroids made them lose their job or have to give up their business. The loss of income severely impacted their ability to do housework and pay for their children’s school fees.

“I do not have a job. I used to work in an orphanage and a restaurant. I worked in the morning and in the evening. I had to quit both jobs because of the fibroid.”

-Unemployed woman with uterine fibroids from Port-Au-Prince.

“The disease [uterine fibroids] affects my mother because she would have worked in order to take care of my education. The disease [uterine fibroids] affects my mother because she cannot cook, clean up the house, send me to school.”

-Family member, daughter from Mirebalais.

## Discussion

**Fig. 4:**
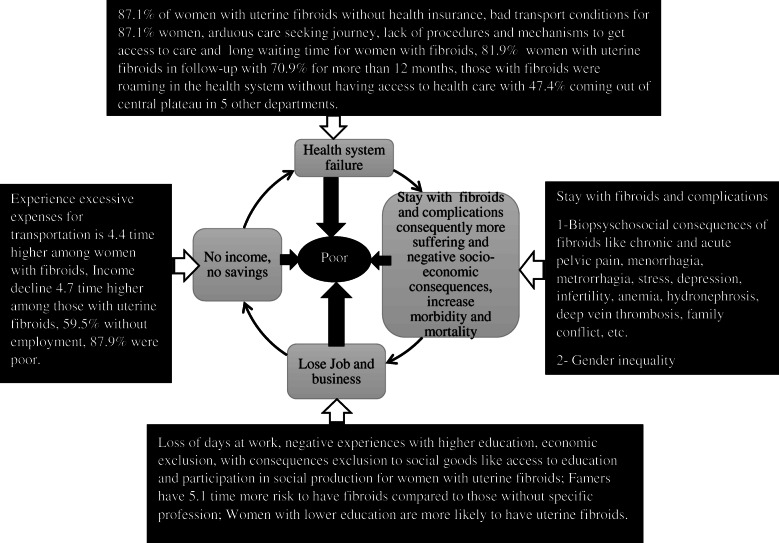
Joint display results: the poverty cycle of uterine fibroids suffering

The study found the quantitative and the qualitative results are interconnected, and we merged them under the concept of a poverty cycle of uterine fibroids (Fig. 4). In the poverty cycle of uterine fibroids one aspect interacts with another. The concept suggests that limited funding constrains the capacity of the health system to deliver care for fibroids because first 87.1% of women with uterine fibroids did not have health insurance that can facilitate access to care and prevent out pocket expenses, second these women roamed in the health system without access to adequate care due the lack of a good ambulance network, procedures and mechanisms that can facilitate access to care, and third 47.1% of these women were coming outside of the catchment area of the hospital in 5 other departments and 70.9% of the participants in follow up for more than 12 months. Consequently, uterine fibroids become complicated and debilitating, and living with untreated fibroids in the long term resulted in possible increases in morbidity and mortality Therefore, women lost their job and business and did not have income and savings, falling deeper into poverty whereas they experienced excess.

The study revealed a high prevalence of uterine fibroids at the MUH’s OPD. This high prevalence was expected because the population had arrived after attempting to receive adequate care at other facilities and the majority of women in the study were between 35 and 49 years of age – the age bracket with highest prevalence of uterine fibroids. Further, ultrasound was used as the reference method to select our population and it has high sensitivity for the diagnosis of uterine fibroid [32].

The study found the most frequently reported symptom was stress. We believe that gender inequity and the complicated uterine fibroids can increase the level of stress among these women. The most two frequent complications reported in our study were anemia and infertility. The finding about anemia is understandable because of the bleeding associated with uterine fibroids. In addition to the presence of fibroids, the lack of specialized care for infertility in the country could be another cause of the high rate infertility observed in this study.

The study did not find an association between age and uterine fibroids in the multiple logistic regression model. This finding is at odds with a previous study which demonstrated that people greater or equal to 40 years old were more likely to have uterine fibroids [1]. In addition, this study demonstrated that health insurance coverage in Haiti was extremely low and was about 6 to 7 times lower than the health insurance coverage in U.S. and Europe [33]. The high out-of-pocket expenditures may be explained by the lack of public health insurance in Haiti or persistent gaps in universal health coverage. As such, women often cannot afford care in a private clinic. Approximately 88%of women with uterine fibroids were poor and 59.5% were unemployed. Our findings suggest that most women with uterine fibroids were waiting for surgical intervention for more than 12 months.

In this study women with uterine fibroids experienced decline in their income after the diagnosis of uterine fibroids, and more than four-fifths of them were poor. These findings can explain why women with uterine fibroids were more likely to experience excessive expenses for transportation to go to the hospital – as even small out of pocket expenditures endangered their ability to provide for their basic needs. The qualitative findings have shown that that women with uterine fibroids continue to endure the disease and its complications, lost their job, and those with lower education were more likely to have uterine fibroids. These findings support the fact that women with fibroids were subjected to economic and social exclusion and poverty. Previous studies have shown that women with uterine fibroids were more likely to miss days at work and lost their job, and those with lower income were more likely to experience severe disease and had negative experiences with higher education [6, 34, 35]. In our study, women with higher education appear to be more likely to seek care, perhaps because women with higher levels of education tend to have more economic power compared to women with lower levels of education.

Our quantitative findings support the concept that women with fibroids were excluded from various forms of ‘social goods’ – most notably health care and education. The qualitative findings provided a deeper and more comprehensive view of the mechanisms by which women with fibroids were socially isolated and were often cast aside by community members or in-laws because their fibroids prevented them from conceiving. Our study underscored the importance of social determinants of health which were contributing factors in women’s suffering from fibroids and supported the emerging concept of the poverty cycle of uterine fibroids.

In the study, women with micro polycystic ovary were less likely to have uterine fibroids whereas other previous studies have shown the contrary [1, 36]. A previous study realized by Huang et al. among women with infertility has shown results in the same direction [37] However the population of this study was different from our population but this similarity can be linked to the high rate of infertile women in our population. In addition, previous studies have shown that family history is an important risk factor for uterine fibroids in the literature [1, 38] and this study also demonstrated this association. However, stress, anemia, depression, menorrhagia, metrorrhagia, dysmenorrhea were not associated with fibroids, whereas a previous study has shown a significant association between these variables and fibroids [6]. The absence of associations of uterine fibroids between stress and anemia can be explained by the fact both are broadly distributed in the population under study. Further study is needed to examine the burden of uterine fibroids among the Haitian population overall.

In term of strengths, this study is the first of its kind in Haiti. It offers important insights into the myriad social and economic effects that women living with uterine fibroids face in settings where access to care is limited. It used ultrasound as the main method to diagnose uterine fibroids with less possibility to miss the diagnosis compared to studies which used self-report questionnaire.

However, this study has several limitations. As a cross-sectional study using a hospital-based population, it is not generalizable to those who have not access care or who have accessed services at a health center. The qualitative data is also not generalizable. It is possible that other clinical conditions may have been misdiagnosed as fibroids - a possible confounder resulting in an overestimation of prevalence. To mitigate misdiagnosis, we used ultrasound which has high specificity and sensibility to establish the diagnosis of uterine fibroids [32]. The prevalence may also be underestimated since uterine fibroids can be also asymptomatic. Further studies are needed to explore the mechanism by which social economic and biological factors increase the risk of uterine fibroids in Haiti. There were no noticeable changes in the study protocol overtime, but we recognize we did the study in the context of a current strike in the country at this period.

## Conclusion

There is a high prevalence of uterine fibroids in our study population. Social and economic consequences associated with fibroids are deeply rooted in a vicious cycle of poverty, gender inequity, physical and social suffering. Although, women are motivated to seek care, they encounter costly delays as they navigate a health care system that is fragmented and overwhelmed by a dearth of equipment and qualified health professionals. Health insurance, availability of biomedical equipment, well trained human resources, expanded infrastructure, and interventions that actively redresses the fragile economic and social conditions facing women with fibroids are needed to promote social and economic rights and improve the health and quality of life of women with uterine fibroids in Haiti.

## Data Availability

The datasets generated and/or analyzed during the current study are not publicly available due to important sensible confidential information about the participants of the study but are available from the corresponding author on reasonable request.

## Abbreviations

MUH: Mirebalais University Hospital,
OPD: Out-Patient Department
AOR: Adjusted Odds Ratio
OR: Odds Ratio
ZL-DSI: Zanmi Lasante Depression Symptom Inventory
OB-GYN: Obstetrics and Gynecology
GYN: Gynecology
